# 4-(3,5-Dimethyl-1*H*-pyrazol-1-yl)benzene­sulfonamide

**DOI:** 10.1107/S1600536811032867

**Published:** 2011-08-27

**Authors:** Abdullah M. Asiri, Hassan M. Faidallah, Abdulrahman O. Al-Youbi, Seik Weng Ng

**Affiliations:** a Chemistry Department, Faculty of Science, King Abdulaziz University, PO Box 80203 Jeddah, Saudi Arabia; b Center of Excellence for Advanced Materials Research, King Abdulaziz University, PO Box 80203 Jeddah, Saudi Arabia; c Department of Chemistry, University of Malaya, 50603 Kuala Lumpur, Malaysia

## Abstract

The two aromatic rings of the title compound, C_11_H_13_N_3_O_2_S, are inclined at an angle of 47.81 (4)°. The N atom of the amino unit is pyramidally coordinated; one H atom inter­acts with the sulfamyl O atom of an adjacent mol­ecule, forming a centrosymmetric hydrogen-bonded dimer. The dimers are linked by N—H⋯N hydrogen bonds, generating a three-dimensional network.

## Related literature

For the synthesis and medicinal properties of the title compound, see: Grueneberg *et al.* (2002 ▶); Wright *et al.* (1964 ▶).
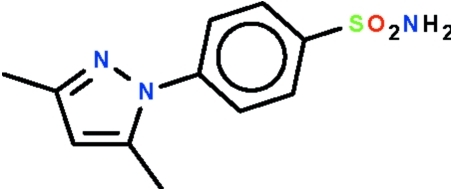


## Experimental

### 

#### Crystal data

C_11_H_13_N_3_O_2_S
*M* *_r_* = 251.30
Monoclinic, 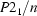

*a* = 7.9649 (1) Å
*b* = 11.7827 (2) Å
*c* = 12.2720 (2) Å
β = 91.720 (1)°
*V* = 1151.18 (3) Å^3^
*Z* = 4
Cu *K*α radiation
μ = 2.47 mm^−1^
*T* = 100 K
0.30 × 0.20 × 0.02 mm

#### Data collection

Agilent SuperNova Dual diffractometer with an Atlas detector
Absorption correction: multi-scan (*CrysAlis PRO*; Agilent, 2010 ▶) *T*_min_ = 0.525, *T*_max_ = 0.952
8510 measured reflections
2312 independent reflections
2215 reflections with *I* > 2σ(*I*)
*R*_int_ = 0.018

#### Refinement

*R*[*F* ^2^ > 2σ(*F* ^2^)] = 0.031
*wR*(*F* ^2^) = 0.083
*S* = 1.07
2312 reflections
164 parameters
2 restraints
H atoms treated by a mixture of independent and constrained refinement
Δρ_max_ = 0.35 e Å^−3^
Δρ_min_ = −0.41 e Å^−3^

### 

Data collection: *CrysAlis PRO* (Agilent, 2010 ▶); cell refinement: *CrysAlis PRO*; data reduction: *CrysAlis PRO*; program(s) used to solve structure: *SHELXS97* (Sheldrick, 2008 ▶); program(s) used to refine structure: *SHELXL97* (Sheldrick, 2008 ▶); molecular graphics: *X-SEED* (Barbour, 2001 ▶); software used to prepare material for publication: *publCIF* (Westrip, 2010 ▶).

## Supplementary Material

Crystal structure: contains datablock(s) global, I. DOI: 10.1107/S1600536811032867/bt5610sup1.cif

Structure factors: contains datablock(s) I. DOI: 10.1107/S1600536811032867/bt5610Isup2.hkl

Supplementary material file. DOI: 10.1107/S1600536811032867/bt5610Isup3.cml

Additional supplementary materials:  crystallographic information; 3D view; checkCIF report

## Figures and Tables

**Table 1:** Hydrogen-bond geometry (Å, °)

*D*—H⋯*A*	*D*—H	H⋯*A*	*D*⋯*A*	*D*—H⋯*A*
N3—H1⋯O1^i^	0.87 (1)	2.13 (1)	2.966 (2)	160 (2)
N3—H1⋯N2^ii^	0.87 (1)	2.94 (2)	3.501 (2)	124 (2)

Symmetry codes: (i) 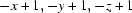; (ii) 
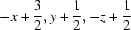
.

**Fig. 1.**
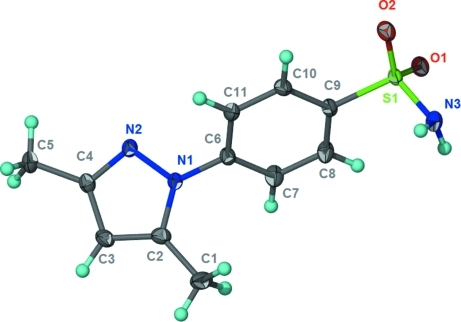
Anisotropic displacement ellipsoid plot (Barbour, 2001) of C11H13N3O2S at the 70% probability level; hydrogen atoms are drawn as spheres of arbitrary radius.

## supplementary crystallographic information

### Comment

The title compound (Scheme I) was first synthesized in order to examine its anti-diabetic activity (Wright *et al.*, 1964). It has also been listed in a virtual screening of compound libraries in order to search for possible medicinal properties (Grueneberg *et al.*, 2002). The two aromatic rings are inclined at 47.81 (4) °. The N atom of the amino unit is pyramidally coordinated (Fig. 1). One H atom interacts with the sulfamyl O atom of an adjacent molecule to form a centrosymmetric hydrogen-bonded dimer; the dimers are linked by an N–H···N hydrogen bond to generate a layer motif.

### Experimental

2,4-Pentanedione (10 mmol) and 4-hydrazinobenzenesulfonamide hydrochloride (10 mmol) were heated in ethanol (50 ml) for 4 h; water was then added to precipitate the product. This was collected and recrystallized from ethanol to yield orange crystals; m.p. 516–517 K.

### Refinement

Carbon bound H-atoms were placed in calculated positions [C–H 0.95 to 0.98 Å, *U*_iso_(H) 1.2–1.5*U*_eq_(C)] and were included in the refinement in the riding model approximation.

The amino H-atoms were located in a difference Fouier map and were refined with a distance restraint of N–H 0.88±0.01 Å; their displacement parameters   were freely refined.

### Crystal data

**Table:**

C_11_H_13_N_3_O_2_S	*F*(000) = 528
*M**_r_* = 251.30	*D*_x_ = 1.450 Mg m^−^^3^
Monoclinic, *P*2_1_/*n*	Cu *K*α radiation, λ = 1.54184 Å
Hall symbol: -P 2yn	Cell parameters from 6177 reflections
*a* = 7.9649 (1) Å	θ = 3.6–74.0°
*b* = 11.7827 (2) Å	µ = 2.47 mm^−^^1^
*c* = 12.2720 (2) Å	*T* = 100 K
β = 91.720 (1)°	Plate, orange
*V* = 1151.18 (3) Å^3^	0.30 × 0.20 × 0.02 mm
*Z* = 4	

### Data collection

**Table:**

Agilent SuperNova Dual diffractometer with an Atlas detector	2312 independent reflections
Radiation source: SuperNova (Cu) X-ray Source	2215 reflections with *I* > 2σ(*I*)
Mirror	*R*_int_ = 0.018
Detector resolution: 10.4041 pixels mm^-1^	θ_max_ = 74.2°, θ_min_ = 5.2°
ω scans	*h* = −7→9
Absorption correction: multi-scan (*CrysAlis PRO*; Agilent, 2010)	*k* = −14→14
*T*_min_ = 0.525, *T*_max_ = 0.952	*l* = −15→15
8510 measured reflections	

### Refinement

**Table:**

Refinement on *F*^2^	Primary atom site location: structure-invariant direct methods
Least-squares matrix: full	Secondary atom site location: difference Fourier map
*R*[*F*^2^ > 2σ(*F*^2^)] = 0.031	Hydrogen site location: inferred from neighbouring sites
*wR*(*F*^2^) = 0.083	H atoms treated by a mixture of independent and constrained refinement
*S* = 1.07	*w* = 1/[σ^2^(*F*_o_^2^) + (0.0441*P*)^2^ + 0.7227*P*] where *P* = (*F*_o_^2^ + 2*F*_c_^2^)/3
2312 reflections	(Δ/σ)_max_ = 0.001
164 parameters	Δρ_max_ = 0.35 e Å^−^^3^
2 restraints	Δρ_min_ = −0.41 e Å^−^^3^

### Fractional atomic coordinates and isotropic or equivalent isotropic displacement parameters (Å^2^)

**Table:**

	*x*	*y*	*z*	*U*_iso_*/*U*_eq_	
S1	0.60099 (4)	0.32597 (3)	0.50334 (2)	0.01314 (12)	
O1	0.44016 (12)	0.37270 (8)	0.52982 (8)	0.0170 (2)	
O2	0.65114 (14)	0.21872 (9)	0.54890 (8)	0.0208 (2)	
N1	0.61599 (14)	0.27786 (10)	0.02059 (9)	0.0136 (2)	
N2	0.55511 (14)	0.18062 (10)	−0.02758 (9)	0.0143 (2)	
N3	0.74013 (15)	0.41830 (10)	0.54060 (9)	0.0152 (2)	
C1	0.7815 (2)	0.45338 (13)	−0.02125 (12)	0.0232 (3)	
H1A	0.8510	0.4777	−0.0815	0.035*	
H1B	0.8535	0.4386	0.0434	0.035*	
H1C	0.7007	0.5132	−0.0049	0.035*	
C2	0.68933 (17)	0.34759 (12)	−0.05284 (11)	0.0161 (3)	
C3	0.67052 (18)	0.29522 (12)	−0.15234 (11)	0.0170 (3)	
H3	0.7068	0.3228	−0.2205	0.020*	
C4	0.58685 (17)	0.19256 (12)	−0.13311 (11)	0.0147 (3)	
C5	0.53563 (19)	0.10247 (13)	−0.21321 (11)	0.0196 (3)	
H5A	0.4668	0.0456	−0.1771	0.029*	
H5B	0.6361	0.0660	−0.2413	0.029*	
H5C	0.4705	0.1367	−0.2737	0.029*	
C6	0.61070 (16)	0.28964 (12)	0.13566 (11)	0.0136 (3)	
C7	0.55663 (18)	0.39093 (12)	0.18128 (11)	0.0183 (3)	
H7	0.5220	0.4521	0.1355	0.022*	
C8	0.55342 (18)	0.40237 (12)	0.29364 (11)	0.0179 (3)	
H8	0.5176	0.4715	0.3253	0.021*	
C9	0.60328 (17)	0.31154 (11)	0.35950 (11)	0.0137 (3)	
C10	0.65597 (18)	0.21010 (12)	0.31423 (11)	0.0163 (3)	
H10	0.6891	0.1485	0.3600	0.020*	
C11	0.66000 (18)	0.19918 (12)	0.20165 (11)	0.0163 (3)	
H11	0.6963	0.1302	0.1700	0.020*	
H1	0.712 (2)	0.4870 (10)	0.5225 (15)	0.029 (5)*	
H2	0.8410 (14)	0.3966 (16)	0.5246 (16)	0.029 (5)*	

### Atomic displacement parameters (Å^2^)

**Table:**

	*U*^11^	*U*^22^	*U*^33^	*U*^12^	*U*^13^	*U*^23^
S1	0.01682 (19)	0.01279 (19)	0.00991 (18)	0.00155 (11)	0.00214 (12)	−0.00074 (11)
O1	0.0163 (5)	0.0191 (5)	0.0160 (5)	0.0001 (4)	0.0045 (4)	−0.0025 (4)
O2	0.0332 (6)	0.0157 (5)	0.0138 (5)	0.0049 (4)	0.0028 (4)	0.0018 (4)
N1	0.0153 (6)	0.0143 (6)	0.0110 (5)	−0.0028 (4)	−0.0002 (4)	−0.0017 (4)
N2	0.0142 (5)	0.0159 (6)	0.0128 (6)	−0.0034 (4)	0.0002 (4)	−0.0028 (4)
N3	0.0144 (6)	0.0161 (6)	0.0150 (6)	0.0024 (4)	−0.0003 (4)	−0.0027 (4)
C1	0.0300 (8)	0.0207 (7)	0.0187 (7)	−0.0104 (6)	−0.0052 (6)	0.0036 (6)
C2	0.0169 (6)	0.0165 (7)	0.0149 (6)	−0.0024 (5)	−0.0014 (5)	0.0036 (5)
C3	0.0194 (7)	0.0199 (7)	0.0117 (6)	−0.0022 (5)	−0.0005 (5)	0.0028 (5)
C4	0.0141 (6)	0.0183 (7)	0.0114 (6)	0.0002 (5)	−0.0011 (5)	−0.0011 (5)
C5	0.0248 (7)	0.0209 (7)	0.0130 (6)	−0.0018 (6)	−0.0006 (5)	−0.0039 (5)
C6	0.0123 (6)	0.0173 (7)	0.0113 (6)	−0.0025 (5)	0.0001 (5)	−0.0018 (5)
C7	0.0222 (7)	0.0173 (7)	0.0151 (7)	0.0050 (5)	−0.0033 (5)	0.0003 (5)
C8	0.0219 (7)	0.0160 (7)	0.0157 (7)	0.0048 (5)	−0.0012 (5)	−0.0033 (5)
C9	0.0136 (6)	0.0159 (6)	0.0117 (6)	−0.0005 (5)	0.0012 (5)	−0.0015 (5)
C10	0.0221 (7)	0.0130 (6)	0.0140 (6)	0.0014 (5)	0.0032 (5)	0.0016 (5)
C11	0.0213 (7)	0.0128 (6)	0.0148 (7)	0.0000 (5)	0.0039 (5)	−0.0020 (5)

### Geometric parameters (Å, °)

**Table:**

S1—O2	1.4338 (10)	C3—H3	0.9500
S1—O1	1.4404 (10)	C4—C5	1.4952 (19)
S1—N3	1.6094 (12)	C5—H5A	0.9800
S1—C9	1.7741 (14)	C5—H5B	0.9800
N1—C2	1.3638 (18)	C5—H5C	0.9800
N1—N2	1.3713 (15)	C6—C11	1.3881 (19)
N1—C6	1.4208 (17)	C6—C7	1.392 (2)
N2—C4	1.3344 (18)	C7—C8	1.3864 (19)
N3—H1	0.869 (9)	C7—H7	0.9500
N3—H2	0.871 (9)	C8—C9	1.3918 (19)
C1—C2	1.4920 (19)	C8—H8	0.9500
C1—H1A	0.9800	C9—C10	1.3882 (19)
C1—H1B	0.9800	C10—C11	1.3889 (19)
C1—H1C	0.9800	C10—H10	0.9500
C2—C3	1.372 (2)	C11—H11	0.9500
C3—C4	1.404 (2)		
			
O2—S1—O1	119.22 (6)	N2—C4—C5	120.48 (12)
O2—S1—N3	107.68 (6)	C3—C4—C5	128.50 (13)
O1—S1—N3	106.68 (6)	C4—C5—H5A	109.5
O2—S1—C9	107.00 (6)	C4—C5—H5B	109.5
O1—S1—C9	107.27 (6)	H5A—C5—H5B	109.5
N3—S1—C9	108.66 (6)	C4—C5—H5C	109.5
C2—N1—N2	111.80 (11)	H5A—C5—H5C	109.5
C2—N1—C6	128.61 (11)	H5B—C5—H5C	109.5
N2—N1—C6	119.31 (11)	C11—C6—C7	120.61 (12)
C4—N2—N1	104.80 (11)	C11—C6—N1	119.24 (12)
S1—N3—H1	112.6 (13)	C7—C6—N1	120.15 (12)
S1—N3—H2	111.7 (13)	C8—C7—C6	119.90 (13)
H1—N3—H2	116.9 (18)	C8—C7—H7	120.0
C2—C1—H1A	109.5	C6—C7—H7	120.0
C2—C1—H1B	109.5	C7—C8—C9	119.29 (13)
H1A—C1—H1B	109.5	C7—C8—H8	120.4
C2—C1—H1C	109.5	C9—C8—H8	120.4
H1A—C1—H1C	109.5	C10—C9—C8	120.94 (13)
H1B—C1—H1C	109.5	C10—C9—S1	119.54 (10)
N1—C2—C3	106.24 (12)	C8—C9—S1	119.52 (10)
N1—C2—C1	123.29 (13)	C9—C10—C11	119.61 (13)
C3—C2—C1	130.28 (13)	C9—C10—H10	120.2
C2—C3—C4	106.11 (12)	C11—C10—H10	120.2
C2—C3—H3	126.9	C6—C11—C10	119.64 (13)
C4—C3—H3	126.9	C6—C11—H11	120.2
N2—C4—C3	111.01 (12)	C10—C11—H11	120.2
			
C2—N1—N2—C4	−1.97 (15)	C11—C6—C7—C8	0.7 (2)
C6—N1—N2—C4	−176.38 (11)	N1—C6—C7—C8	−179.31 (12)
N2—N1—C2—C3	1.89 (15)	C6—C7—C8—C9	−0.5 (2)
C6—N1—C2—C3	175.64 (13)	C7—C8—C9—C10	0.0 (2)
N2—N1—C2—C1	−173.51 (13)	C7—C8—C9—S1	179.52 (11)
C6—N1—C2—C1	0.2 (2)	O2—S1—C9—C10	−2.85 (13)
N1—C2—C3—C4	−1.01 (15)	O1—S1—C9—C10	−131.88 (12)
C1—C2—C3—C4	173.95 (15)	N3—S1—C9—C10	113.15 (12)
N1—N2—C4—C3	1.28 (15)	O2—S1—C9—C8	177.63 (11)
N1—N2—C4—C5	−179.21 (12)	O1—S1—C9—C8	48.60 (13)
C2—C3—C4—N2	−0.18 (16)	N3—S1—C9—C8	−66.37 (13)
C2—C3—C4—C5	−179.65 (14)	C8—C9—C10—C11	0.4 (2)
C2—N1—C6—C11	−128.45 (15)	S1—C9—C10—C11	−179.13 (11)
N2—N1—C6—C11	44.90 (17)	C7—C6—C11—C10	−0.3 (2)
C2—N1—C6—C7	51.5 (2)	N1—C6—C11—C10	179.71 (12)
N2—N1—C6—C7	−135.12 (13)	C9—C10—C11—C6	−0.2 (2)

### Hydrogen-bond geometry (Å, °)

**Table:**

*D*—H···*A*	*D*—H	H···*A*	*D*···*A*	*D*—H···*A*
N3—H1···O1^i^	0.87 (1)	2.13 (1)	2.966 (2)	160 (2)
N3—H1···N2^ii^	0.87 (1)	2.94 (2)	3.501 (2)	124 (2)

Symmetry codes: (i) −*x*+1, −*y*+1, −*z*+1; (ii) −*x*+3/2, *y*+1/2, −*z*+1/2.
